# Benign Oesophageal Stricture and Chronic Diarrhoea As Atypical Presenting Symptoms of an Advanced Metastatic Pancreatic Gastrinoma: A Case Report and Review of Literature

**DOI:** 10.7759/cureus.16593

**Published:** 2021-07-23

**Authors:** Muhammad Hafiz Kamarul Bahrin, Raoof Hagag, Abuobeida Ali, Seifeldin Yahia, Richard Armstrong

**Affiliations:** 1 Gastroenterology, United Lincolnshire Hospitals Trust, Boston, GBR; 2 Acute Medicine, United Lincolnshire Hospitals National Health Service (NHS) Trust, Boston, GBR; 3 Gastroenterology, Peterborough City Hospital, Peterborough, GBR; 4 Diabetes and Endocrinology, United Lincolnshire Hospitals National Health Service (NHS) Trust, Boston, GBR; 5 Gastroenterology, United Lincolnshire Hospitals National Health Service (NHS) Trust, Boston, GBR

**Keywords:** gastrinoma, zollinger-ellison syndrome, benign oesophageal stricture, chronic diarrhoea, pancreatic neuroendocrine tumours

## Abstract

Gastrinoma or otherwise known as Zollinger-Ellison syndrome is characterised by hypersecretion of gastrin and gastric acid leading to the formation of recurrent atypical ulcers along the upper gastrointestinal tract. It is extremely difficult to diagnose during an acute presentation both due to its rarity and its lack of pathognomonic symptoms. Its symptoms range from mild to severe to life-threatening and often get mistaken for a different condition such as viral gastroenteritis as seen in our case report. The most common symptoms of gastrinoma include abdominal pain, dyspepsia and chronic diarrhoea. It rarely presents as a benign oesophageal stricture with some case series reporting the frequency to be as low as 0.4%. Our literature review of 9 random case reports on gastrinoma/Zollinger-Ellison syndrome selected from Pubmed Central reviewed the frequency of its presenting symptoms and investigation modalities involved throughout its diagnostic process. In summary, it agrees with the findings postulated by Jensen’s series. We also looked into the use of Ga68- DOTATATE-PET/CT as the latest imaging modality used in diagnosing and staging gastrinoma. Once suspected, it is imperative for physicians to investigate it through laboratory, radio-imaging, histology and multidisciplinary led investigating approaches. Depending on its stage, treatment options vary. Early and localised gastrinoma cases are often treated surgically whilst metastasised cases usually resort to treatment with palliative intent.

## Introduction

Gastrinoma is a pancreatic neuroendocrine tumour (p-NET). It is extremely challenging to diagnose in an acute setting both due to its rarity and its lack of pathognomonic symptoms. As such, they are usually only diagnosed after years of symptom presentation. Its incidence is between 0.5 and 15 cases per million population and generally accounts for 0.1% gastric ulcer and 2%-5% recurrent ulcers [1]. Gastrinoma is also referred to as Zollinger-Ellison syndrome (ZES), which is a condition characterised by gastrin hypersecretion, gastro-oesophageal hyperacidity and recurrent atypical ulcers as described by Zollinger and Ellison in 1955 [1]. Gastrinoma is staged according to the standard World Health Organization (WHO) tumour, nodes and metastases (TNM) staging system, which consequently determines its treatment options. Generally, early gastrinoma is treated with curative resection. Liver involvement, with the exception of primary hepatic gastrinoma, indicates poor prognosis and is usually treated conservatively.

There are other forms of p-NET and they are classified generally as functional and non-functional p-NET. The 2012 Consensus Statement by the European Neuroendocrine Tumour Society (ENETS) stated that the two most common functional, malignant p-NETs are gastrinoma and insulinoma. Rarer functional p-NETs account for less than 0.2 cases/million population/year and they include glucagonoma, vasoactive intestinal peptide + oma (VIP-oma), somatostatinoma, GH-oma and ACTH-oma. Very rare functional p-NETs are still under investigation and their incidence remains unknown - they include p-NET secreting PTHrp (PTHrp-oma) and p-NET causing serotonin-induced carcinoid syndrome. Non-functional p-NETs often secrete a pancreatic polypeptide, chromogranin A, neuron-specific enolase, human chorionic gonadotrophin subunits, calcitonin, neurotensin or other peptides, but they do not result in clinically significant symptoms manifestation - hence they are largely considered non-functional tumours [2].

We report a case of gastrinoma in an elderly English lady, presenting as recurrent abdominal pain, vomiting and diarrhoea being initially mistaken as a series of acute viral gastroenteritis. Following her admission with worsening symptoms, oesophago-gastro-duodenoscopy (OGD) revealed an extensive oesophago-gastritis, benign distal oesophageal stricture and atypical duodenal ulcers, raising suspicion of an underlying gastrinoma. Radio-imaging investigations, histopathology and multi-disciplinary diagnostic meetings confirmed the diagnosis of a pancreatic head gastrinoma with liver metastases. Our patient received conservative management with a good outcome.

## Case presentation

We report a 79 years old English lady who presented in July 2017 with a complaint of abdominal pain and repeated vomiting for a week. She also reported occasional non-bloody watery diarrhoea over the past one month. She experienced similar symptoms back in January and was diagnosed with acute viral gastroenteritis. Her past medical history includes well-controlled hypertension for which she takes regular amlodipine 5 mg once a day (OD). She does not take any regular medication apart from this. Her family history was unremarkable. On her social history, she denied smoking and drinking alcohol. Physical examination demonstrated stable observation parameters. Clinical biochemistry blood tests showed mild derangement of the liver function test (LFT) with elevated alanine transaminase (ALT) at 64 U/L (normal range 7-55 U/L) and alkaline phosphatase (ALP) at 329 U/L (normal range 40-129 U/L). She was treated with intravenous (IV) cyclizine 50 mg as a once-off dose and IV 0.9% sodium chloride at 125 mL/hour for 24 hours. After three days, her symptoms resolved and she was discharged home with a provisional diagnosis of another acute viral gastroenteritis.

After 13 days following the discharge from the hospital, she re-attended the Emergency Department (ED) with worsening symptoms in addition to a new-onset dysphagia and complete loss of appetite for five days. She also complained of a new-onset watery non-bloody diarrhoea. Physical examination revealed a mildly tender epigastrium without any sign of peritonism. At this point, her haematology and biochemistry blood tests were as shown in Table 1. She received IV 0.9% sodium chloride 125 mL/hour, IV cyclizine 50 mg three times a day (TDS) and IV paracetamol 1 g four times a day (QDS).

**Table 1:** Blood test results during the patient’s second admission. Note that patient was considerably anaemic given no recent history of bleeding or dietary issue. This may be the first clue pointing towards an underlying malignancy.

Blood investigation	Result	Normal range
Full Blood Count (FBC)	94	140–170 g/L
White Cell Count (WCC)	9.5	4.5–11 x 109 cells/L
Platelet	507	150–350 x 109/L
Total Bilirubin (Bil)	6	3-17 micromol/L
Alanine Transaminase (ALT)	33	5-35 IU/L
Aspartate Transaminase (AST)	258	5-40 IU/L
Alkaline Phosphatase (ALP)	156	30-150 IU/L
Albumin (Alb)	27	35-50 g/L

**Figure 1:**
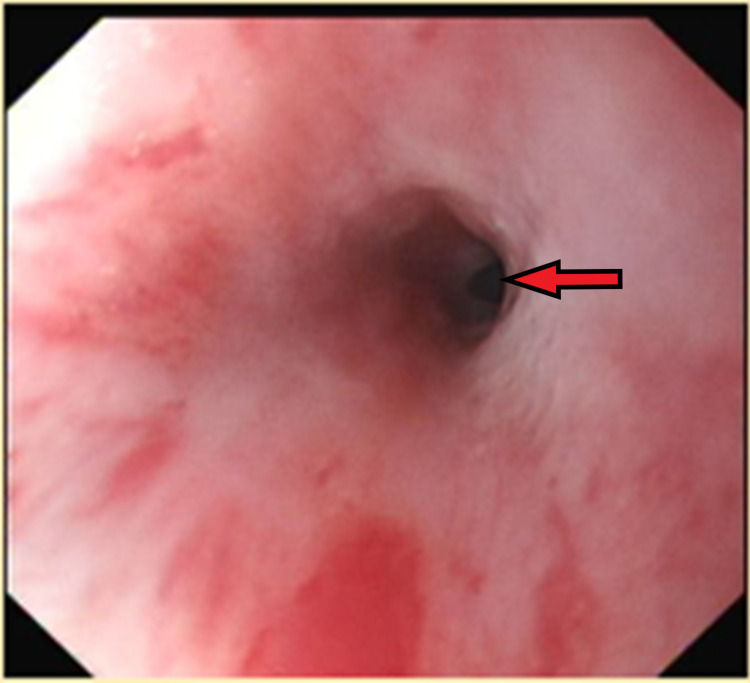
OGD image demonstrating a benign mid-oesophageal stricture OGD - oesophago-gastro-duodenoscopy

**Figure 2:**
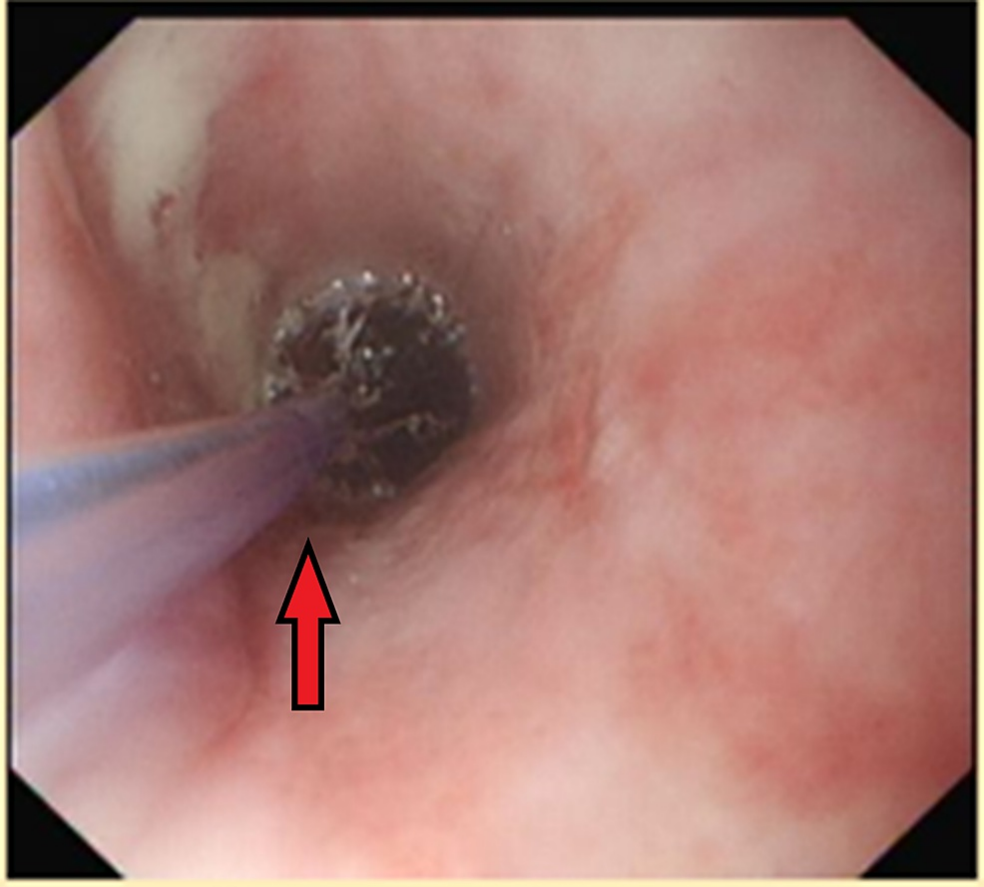
Endoscopic dilatation of the benign oesophageal stricture to 12 mm in diameter using controlled radial expansion (CRE) wire-guided balloon dilatation catheter

**Figure 3:**
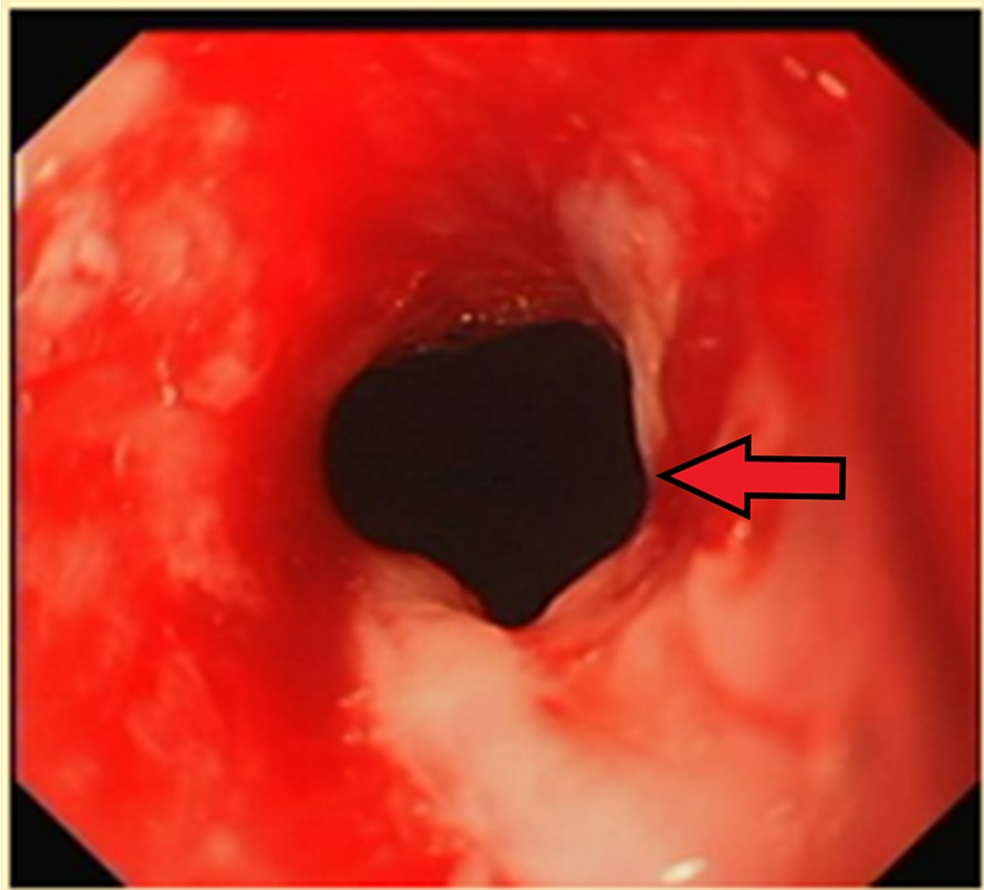
Post dilatation appearance of the benign oesophageal stricture

She underwent an OGD the next day, at which point the diarrhoea had stopped. This demonstrated a benign-looking mid-oeosphageal stricture, gastritis, erosive duodenitis in D1 and multiple ulcers with large, ulcerated mucosa in D2. Figure 1 shows the benign stricture image. Unfortunately, no image was taken for the duodenal ulcers in D2. Figure 2 shows the endoscopic dilatation of the benign oesophageal stricture using a wire-guided balloon dilator. Figure 3 shows the post-dilatation appearance of the stricture. Otherwise, no stigmata of upper gastrointestinal bleeding (UGIB) was noted. The abnormal OGD findings led us to a suspicion of a ZES with an underlying malignancy.

**Figure 4:**
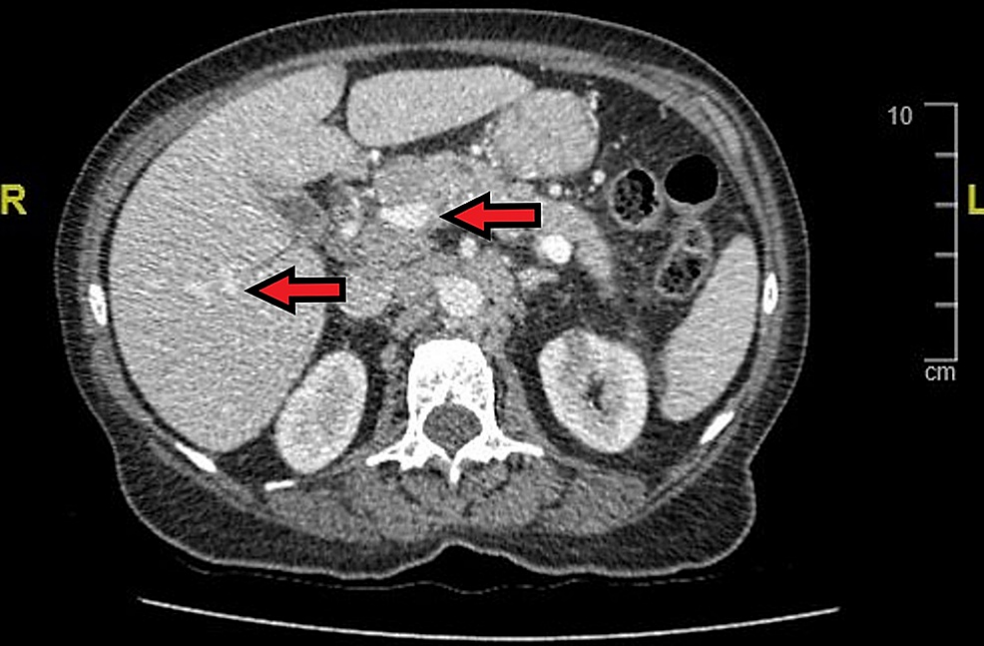
Transverse plane view of patient’s CT abdomen showing gastrinoma mass (arrows) in the pancreatic head with the appearance of metastatic lesions in the liver

**Figure 5:**
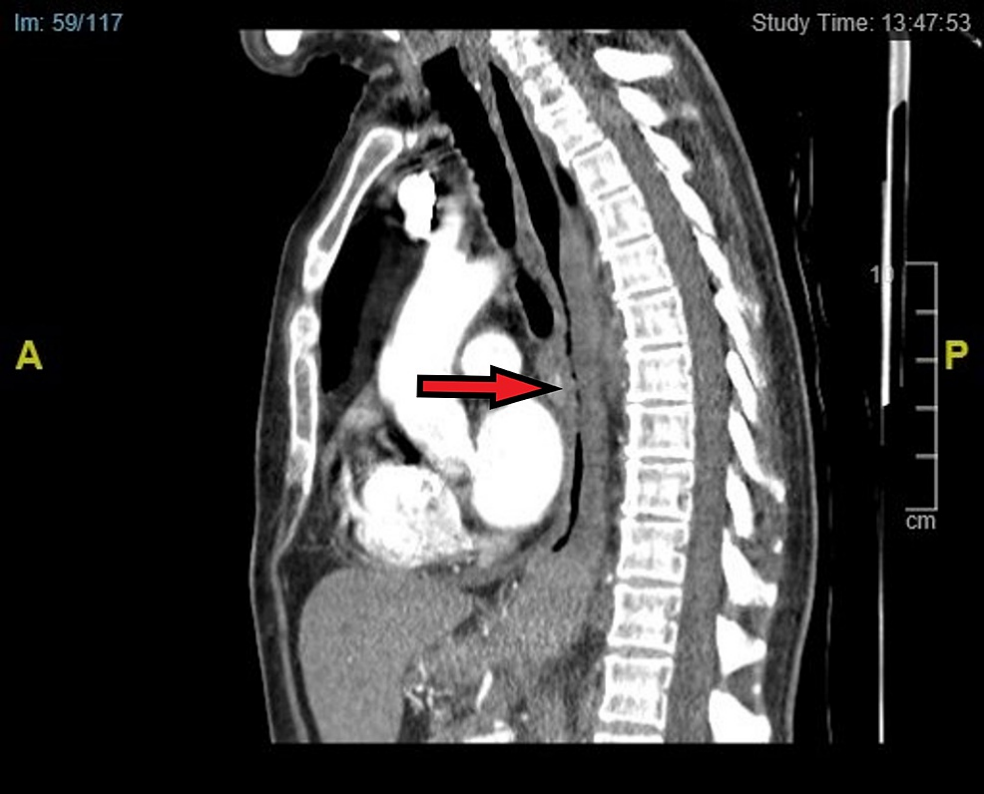
Sagittal plane view of our patient’s CT thorax showing stricturing oesophagus from middle to distal section This correlates with our OGD finding as described in Figure 1.

An investigation was followed up with a full-body contrast-enhanced computed tomography (CT) scan. This demonstrated abnormal appearances of the proximal pancreatic head, multiple enlarged para-aortic nodes and hepatic enhancement, raising suspicion of metastases (Figure 4). There was also indeterminate thickening of the gastro-oesophageal junction (GOJ) in keeping with the OGD finding described above (Figure 5). Common bile duct (CBD) was spared from obstruction and our patient remained non-icteric throughout her admission.

The findings above immediately raised our suspicion of the presence of a neuroendocrine tumour (NET) in the form of gastrinoma. Histology sample was obtained via core needle biopsy of the said liver lesion, which was reported as ‘a well differentiated neuroendocrine neoplasm made of monomorphic cells showing positive stain for chromogranin, synaptophysin, CD56 and AE1/AE3 with up to 25% of tumour cells staining for Ki67’. Unfortunately, the histopathology images could not be obtained. She was discharged with a high-dose oral proton pump inhibitor (PPI) in the form of omeprazole 60 mg twice a day (BD) and oral cyclizine 50 mg TDS.

**Figure 6:**
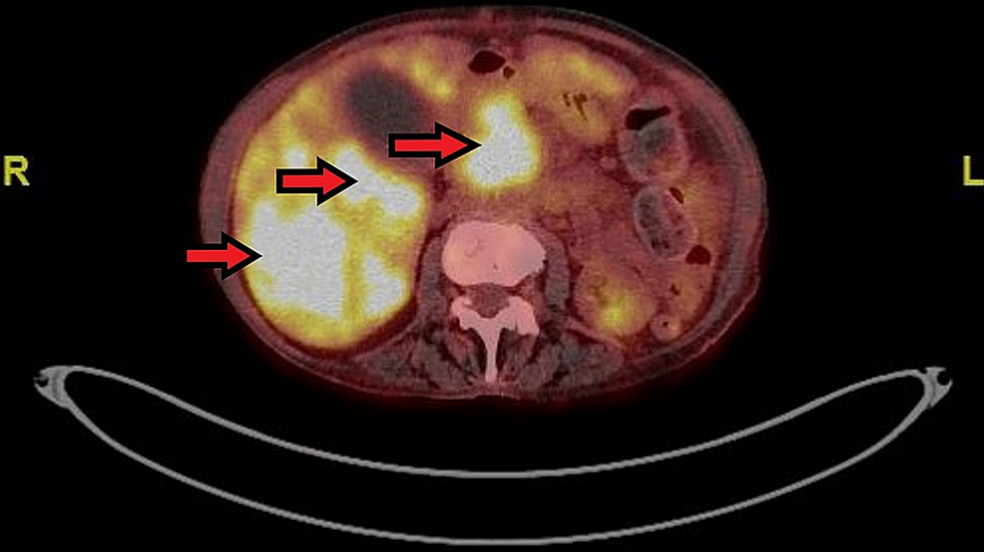
Transverse plane view of patient’s Ga68-DOTATATE-PET/CT scan demonstrating multiple foci of intense increased tracer uptake within both lobes of the liver and within the proximal pancreas, signifying a metastatic NET NET - neuroendocrine tumour

Over the next few months, she underwent a further confirmatory tests that consisted of pancreatic neuroendocrine serology tests as shown in Table 2 and a whole-body Ga68-DOTATATE-PET/CT which was reported in Figure 6. Both investigations confirmed the suspected gastrinoma.

**Table 2:** Pancreatic neuroendocrine serology test results for the patient.

Pancreatic neuroendocrine (p-NET) blood test	Result	Normal range
Chromogranin A	409	0-60 unit/L
Serum Glucagon	<5	0-50 pmol
Serum Pancreatic Polypeptide	>500	0-300 pmol
Serum somatostatin	29	0-150 pmol
Serum fasting gastrin	>400	0-40 pmol
Serum Vasoactive Intestinal Pepide (VIP)	9	0-30 pmol

Following this, she received a monthly IV somatostatin analogue in the form of Lanreotide 120 mg, which was however terminated in early 2019 due to disease progression. She also attended the endoscopy department on a four-monthly basis for palliative endoscopic dilatation of the recurring oesophageal stricture using controlled radial expansion (CRE) wire-guided balloon dilatation catheter to 12 mm in diameter, intended to alleviate her dysphagia. In view of her advanced age and the metastatic nature of her gastrinoma, she was deemed unsuitable for surgical resection of the tumour - which we were aware of as the gold standard treatment for a NET. The NET multidisciplinary team meeting (MDT) reached an agreement that further management of her gastrinoma should be conservative in nature with palliative intent. She is currently still on an ongoing chemotherapy regime consisting of Carboplatin, fluorouracil and streptozocin. Surveillance CT scan of her gastrinoma in early 2020 demonstrated a relatively stable tumour size and stage. Her quality of life remains unchanged. 

## Discussion

Literature review

A quick search on Pubmed Central (https://pubmed.ncbi.nlm.nih.gov/) was carried out with regards to gastrinoma and ZES. Nine case reports describing gastrinoma/ZES in nine different patients were selected at random and analysed in terms of their epidemiology, symptomatology, investigation modalities, presence of metastasis and subsequent treatment options. The findings are tabulated as demonstrated in Table 3.

**Table 3:** A literature review of nine case reports on gastrinoma/Zollinger-Ellison syndrome selected at random from Pubmed Central.

Number	Study, year	Age, gender	Primary symptoms	Imaging modalities used	Presence of metastases	Treatment
1	Alshiko et al., 2016 [3]	28, female	Heartburn, Diarrhoea, Nausea and Vomiting, Weight Loss	OGD, CT abdomen	None	Surgical resection
2	Takami et al., 1978 [4]	46, male	Heartburn	Immunofluorescence studies	None	Surgical resection
3	Aamar et al., 2016 [5]	7, female	Nausea, Vomiting, Diarrhoea, Haematemesis, Abdominal Pain	OGD, CT abdomen, PET scan,	None	High-dose omeprazole and octreotide
4	Baig et al., 2011 [6]	67, male	Frequent nausea, Vomiting, Diarrhoea, Haematemesis, Severe abdominal pain	OGD, Endoscopic Ultrasound (EUS), Octreotide scan, MRI of the abdomen	None	Surgical resection
5	Shah et al., 2019 [7]	60, female	Nausea, Vomiting, Watery diarrhoea, Abdominal pain	OGD, EUS	None	Distal Pancreatectomy High-dose PPI Octreotide
6	Rivillas-Reyes et al., 2019 [8]	42, female	Epigastric pain, Melena Signs of peritoneal irritation	OGD	None	Whipple procedure
7	Eyal et al., 2014 [9]	53, male	Abdominal pain, Diarrhoea, Hypomagnesaemia, UGIB	CT scan (Abdomen), OGD, Positron emission tomography-CT scan using GA 68-DOTANOC	None	Pancreaticoduodenectomy
8	Zhang et al., 2016 [1]	68, female	Intermittent abdominal pain, Watery diarrhoea	OGD, CT Abdomen, MRI Abdomen	None	Pancreaticoduodenectomy
9	Sinagra et al., 2013 [10]	61, female	Nausea and vomiting, Watery diarrhoea	OGD	Liver metastasis	Total parenteral nutrition (TPN) feeding, Endoscopic pneumatic dilation of the esophageal stenosis

The literature review highlights that abdominal pain forms the most common presenting symptom of gastrinoma, followed by heartburn, diarrhoea and vomiting. Four patients developed acute UGIB in response to the underlying gastrinoma. Only one patient reported symptoms of oesophageal stricture, requiring pneumatic balloon dilatation. Expectedly, these findings are in keeping with Jensen’s series [11], which highlighted the rarity of oesophageal stricture as a presenting symptom of gastrinoma. Eight out of nine case reports involved the use of OGD at some point during the investigation period - either as the primary investigation leading to suspicion of gastrinoma or secondary investigation to monitor the disease course. Ga68-DOTATATE-PET/CT was only utilised in only 1 case report, with another one utilising the relatively traditional octreotide scan. Surgical resection was pursued in most non-metastatic gastrinoma cases but for the one case involving liver metastasis, the treatment approach was conservative.

Literature analysis

Gastrinoma is a pancreatic neuroendocrine neoplasm (pNEN) associated with excessive release of gastrin hormone leading to hypersecretion of gastric acid. This subsequently results in hyperacidity of the stomach, recurrent gastro-duodenal ulcers and a minority of cases, severe reflux disease complicating into oesophageal stricture. The vast array of symptoms related to hypersecretion of gastric acid is called Zollinger-Ellison syndrome (ZES). Gastrinoma affects mainly males than females with a prevalence ratio ranging between 1.5:1 and 2:1. It affects people across all age groups, however is predominant between 20 and 60 years old (by up to 90%) as demonstrated in a study by Hoffman et al. [12].

In most cases (up to 80%), gastrinoma presents as sporadic while in the rest 20% of cases, it presents in combination with other NETs under a condition named Multiple Endocrine Neoplasia Type 1 (MEN-1) [12]. MEN-1 generally involves tumour growths in the parathyroid glands (leading to recurrent hypercalcaemia secondary to excessive parathyroid hormone secretion), pituitary glands (leading to oligomenorrhoea/amenorrhoea/galactorrhoea in females and sexual dysfunction in males secondary to prolactinoma) and the pancreatic neuroendocrine cell. The mode of inheritance for MEN-1 is autosomal dominant in most cases. Sporadic gastrinoma, on the other hand, does not exhibit a specific mode of inheritance hence is more challenging to anticipate [13], especially in an acute setting.

Diagnosing gastrinoma in the UK requires a combination of laboratory testing, radio-imaging investigations and histology. Often management of gastrinoma is made via a coordinated multidisciplinary effort, involving the gastroenterologist, endocrinologist, medical oncologist, surgeon, interventional radiologist and pathologist. Once ZES is suspected, i.e., atypical gastrointestinal ulcers in the absence of Helicobacter Pylori and refractory to acid-suppressive medications, the first step is to check the serum fasting gastrin level in order to rule out gastrinoma. Serum fasting gastrin level of >200 pg/mL (NR 100-200 pg/mL) raises the likelihood of gastrinoma. The second step is to rule out other secondary causes of hypergastrinemia such as achlorhydria/hypochlorhydria frequently secondary to PPI or pernicious anaemia. To do this, some centres would carry out gastric pH measurement via endoscopy or nasogastric (NG) tube gastric suctioning while patients are taken off of PPI for seven days. Gastric pH of 1,000 pg/mL is virtually diagnostic for this condition. Similarly, if serum fasting gastrin level increases >100 pg/mL but by 200 pg/mL or twofold above the basal level, this is diagnostic for a primary hypergastrinemia and hence, gastrinoma [14]. Sometimes serum chromogranin A level is also checked; however, this test lacks specificity (67%) despite having high sensitivity (53%-91%) [15].

As with any cancer diagnosis, obtaining a tissue sample is mandatory to determine its grade. This is achieved through core needle biopsy of accessible malignant lesions as visualised on CT or MRI scan. Once obtained, the tissue is stained with Ki67, an immunostaining protein that binds to and picks up proliferating NET cells. Percentage of stained nuclei are then measured and classified into 3 stages. Namely, low-grade NET produces a Ki67 score of 20% as in this case correlates to high-grade NET [13]. CT and MRI scans would help to determine the extent of the spread of the disease. However, the gold standard imaging modality for the diagnosis of gastrinoma is PET/CT with Octreotide Scintigraphy using Gallium-68 (Ga68) or otherwise known as Ga68-DOTATATE-PET/CT. As indicated in its name, it uses a newer radioisotope of Gallium-68, which can be linked to somatostatin analogues, picked up by the tumour cells and localised by PET/CT scan to give a better picture of the extent of tumour spread. A retrospective single-centre study in the US demonstrated that in contrast to the standard somatostatin receptor scintigraphy, Ga68- DOTATATE-PET/CT has a higher sensitivity (90% vs 65%) and overall accuracy (88% vs 68%) [16]. Its use is currently extremely limited in the UK, only available in some tertiary referral centres.

Definitive management of a NET such as gastrinoma is achieved through surgical resection of the said tumour for a localised stage. The 20-year survival rate in a primary gastrinoma without metastasis is as high as 95%. Contrastingly, in metastatic gastrinoma cases such as our patients, especially those that had affected the liver, the prognosis is generally poor and surgical resection is usually not feasible and treatment intent is palliative [17]. However, this is debatable as according to a study by Glazer et al., which suggested that early and aggressive surgical management of hepatic metastases from NETs is associated with significant long-term survival rates [18]. Having said so, surgical debulking may be pursued if the tumour growth is large enough to cause a surrounding compression effect.

The first line systemic therapy if the surgical approach is deemed unsuitable, is through symptomatic control by the means of high dose oral PPI (60 mg daily and can be increased up to a maximum of 120 mg daily in two divided doses) and IV somatostatin analogue infusion [19]. Wire-guided oesophageal balloon dilatation is also useful in alleviating the dysphagia symptom according to some case reports. Chemotherapy selection for gastrinoma with metastasis or locally advanced disease consists of 5-Fu, streptozocin and carboplatin. This combination demonstrated an overall favourable outcome in terms of stable disease state post-treatment in 51% of cases and disease progression in 16% only, according to a British-based 79-patients prospective study by Turner et al. [20].

## Conclusions

Gastrinoma, being an exceptionally rare type of aggressive malignancy, poses constant diagnostic and management challenges for general physicians. It lacks a pathognomonic set of symptoms to prompt an acute physician to consider its presence at initial presentation, especially during a hectic acute take setting. Besides, symptoms associated with the early stage of gastrinoma may also be mild, often masked by widespread prescription of PPI and often being mistaken as acute viral gastroenteritis episodes, as demonstrated in our case report. Authors speculate that the overall impact of the above may potentially lead to delayed diagnosis of gastrinoma, risking narrowing of the available treatment options at the point of diagnosis. The authors also recommend that a worldwide scoring system to risk-stratify patients with potential gastrinoma be established, so that high-risk patients could be investigated at an earlier stage either in the community or in the secondary care setting. Further research or systematic analysis into this subject may be required but not within the remit of this case report. 
